# Distinct Gastrointestinal and Reproductive Microbial Patterns in Female Holobiont of Infertility

**DOI:** 10.3390/microorganisms12050989

**Published:** 2024-05-14

**Authors:** Ana T. Marcos, Maria J. Rus, Victoria Areal-Quecuty, Aurea Simon-Soro, José Manuel Navarro-Pando

**Affiliations:** 1 Unidad de Genética, INEBIR (Instituto para el Estudio de la Biología de la Reproducción Humana), 41001 Sevilla, Spainjose.navarro@inebir.com (J.M.N.-P.); 2 Cátedra de Reproducción y Genética Humana, INEBIR/Universidad Europea del Atlántico (UNEATLANTICO), 39011 Santander, Spain; 3 FUNIBER (Fundación Universitaria Iberoamericana), 08005 Barcelona, Spain; 4 Hospital San Juan de Dios, 41005 Sevilla, Spain; 5 Departamento de Estomatología, Facultad de Odontología, Universidad de Sevilla, 41009 Sevilla, Spain

**Keywords:** microbiome, holobiont, oral, tract, female, infertility, endometriosis

## Abstract

The microbiota is in symbiosis with the human body as a holobiont. Infertility conditions affect the female reproductive tract (FRT) and its resident microbiota. However, a disturbance in homeostasis could influence the FRT and other distal body sites, such as the gastrointestinal tract (GIT). We included 21 patients with endometriosis and other infertility-associated diseases with clinical profiles and biological samples from the FRT (endometrium, endometrial fluid, and vagina), and GIT samples (oral and feces). We performed a 16S rRNA analysis of site-specific microbial communities and estimated diversity metrics. The study found body site-specific microbial patterns in the FRT–GIT. In both study groups, *Lactobacillus* was the most shared Amplicon Sequence Variant (ASV), a precise identifier of microbial sequences, between endometrial and vagina samples. However, shared *Gardnerella* and *Enterobacteriaceae* ASVs were linked to other conditions but not endometriosis. Remarkably, *Haemophilus* was a specific GIT-shared taxon in endometriosis cases. In conclusion, infertility influences distinctly the FRT and GIT microbiomes, with endometriosis showing unique microbial characteristics. We proposed the concept of ‘female holobiont’ as a community that comprises the host and microbes that must maintain overall homeostasis across all body sites to ensure a woman’s health. Insights into these microbial patterns not only advance our understanding of the pathophysiology of infertility but also open new avenues for developing microbe-based therapeutic interventions aimed at restoring microbial balance, thereby enhancing fertility prospects.

## 1. Introduction

The human body contains hundreds of microbial species that evolved as a single superorganism. The microorganisms that live inside the human body play a crucial role in maintaining health through a complementary metabolic repertoire [1]. The human body is thus a holobiont, a complex community composed of the host and the microorganisms living in symbiosis. The holobiont has specific body sites defined as the host–microbiota interaction shaped by environmental conditions. The resident microbial community in the mouth, gut, and vagina differs between body sites and host tissues, immune system, or environment (oxygen, pH, humidity). Consequently, the host’s fitness depends on and cannot be seen independent of its microbiota [2]. Although there are distinctive human niches, the holobiont must balance all the body sites to maintain health. The microbiota loses balance when homeostasis is disrupted, also known as dysbiosis. Increasing evidence considers a chronic inflammatory state to be associated with host–microbial imbalance [3].

Endometriosis is a chronic inflammatory disease in which endometrium-like tissue is found outside the uterus. It is associated with severe chronic pain, dyspareunia, dysmenorrhea, and infertility [4]. Approximately 10–15% of women of reproductive age have endometriosis [5]. Endometriosis is often underdiagnosed and reduces the quality of life of a patient [6]. The pathogenesis of endometriosis is multifactorial, and there are several theories of endometriosis development, but none of them has been able to fully explain this enigmatic disease. However, most theories converge that it is influenced by estrogen metabolism and inflammation [4]. Several studies have provided evidence of altered local and systemic immunity in patients with endometriosis [7,8,9,10]. There is currently growing interest in how the microbiome could modulate endometriosis [11,12] since several studies suggest that the microbiome is altered in women with endometriosis [12,13,14] and that its metabolites can promote the disease [15].

The microbiome has been shown to play a role in the success of infertility treatment outcomes. Concretely, *Lactobacillus* dominance has been positively correlated with embryo implantation, pregnancy, and birth rate in patients who have received in vitro fertilization treatment [16]. The vaginal and endometrial microbiomes appear to be altered in women with gynecological diseases and infertility [17,18,19,20]. The vaginal microbiota of healthy women of reproductive age is primarily composed of *Lactobacillus* species. Their presence contributes to controlling the growth of other microorganisms considered pathogenic by preserving a lower vaginal pH, inhibiting their adherence and the release of antimicrobial metabolites, competing for nutrients, and controlling the local immune response [21]. The endometrial microbiota has a unique bacterial composition that differs from the vaginal microbiota [18]. It has been reported that there is less diversity in the endometrial compared to the vaginal microbiome [22,23].

Communication between the FRT and other organs has been established [24]. Specifically, the GIT and FRT share a bidirectional interaction with themselves and other organs through immune, neural, endocrine, and metabolic pathways [25,26]. The microbiota plays an essential role in communication and systemic health through the release of metabolites [27,28,29,30]. Inflammatory diseases such as endometriosis alter the balance of systemic homeostasis, including the microbiome of local and distant body sites [31]. Therefore, modulation of the microbiota could play a critical role in the development of gynecological diseases and infertility [24].

In this cohort study, we examined the microbiomes in the FRT and the GIT to assess their influence on reproductive function in women experiencing infertility. The objective of the study was to describe the microbiome in patients with endometriosis and compare it with that of other gynecological conditions leading to infertility. Additionally, we explored the relationship between microbiological findings and clinical data, focusing on understanding the complex microbial balance across multiple body sites within a hormonal environment, a dynamic that we define as the ‘female holobiont’.

## 2. Materials and Methods

### 2.1. Study Design

We conducted a cohort study at the Instituto para el Estudio de la Biología de la Reproducción Humana (INEBIR) from December 2021 to April 2022 in which oral, vaginal, and intestinal samples of 21 female patients were collected. The inclusion criteria were patients with fertility problems of reproductive age (18–45 years) and who were in the process of in vitro fertilization (IVF). Exclusion criteria included treatment with antibiotics and/or antifungals within 3 months before first sample collection, severe or uncontrolled bacterial, fungal, or viral infections or any disease or medical condition that threatens patient’s safety, positive for human immunodeficiency virus, Hepatitis B, Hepatitis C, and syphilis, under 18 or over 45 years of age, and pregnant women. This study was carried out according to the Declaration of Helsinki on medical research involving human subjects and the Good Clinical Practice guidelines. It was approved by the Ethics Committee of the Universidad Europea del Atlántico (UNEATLANTICO) on 22 December 2020, No. 43. Written informed consent was obtained from all enrolled patients.

Following the selection of subjects, we then subclassified patients into two clinical groups: (a) endometriosis (confirmed by imaging or laparoscopy [32]) and (b) other infertility-related conditions such as ovarian failure, polyp, erythroplakia, hydrosalpinx, chronic endometritis, and polycystic ovary syndrome.

Information on the general health status of the patients was obtained along with age, systemic parameters, drug history, gynecological diseases, obstetric history, demographic data, and microbial profiles. Medication information was collected for antibacterial, antifungal, immunosuppressive, and contraceptive medications such as allergies, intolerances, and habits.

### 2.2. Sample Collection

All samples were taken between days 14 and 16 of the menstrual cycle. Five samples were taken for each patient as follows: (1) vagina, with the patient in a lithotomy position, a speculum was placed in the vagina to visualize the cervix. External genitalia were cleansed with a saline solution (NaCl 0.9%), and a sterile viscose swab (Deltalab, Barcelona, Spain) was introduced until the posterior fornix was reached and rotated to soak the swab for approximately 1 min. After cleaning the external genital area, a speculum was introduced into the vagina, and a sterile swab was taken from the posterior area rotating 360 degrees for one minute; (2) endometrial fluid, the endometrial fluid sample was obtained after the vaginal fluid sample and before the endometrial biopsy. A flexible catheter was inserted 5–8 cm through the cervix into the ultrasound-guided uterine cavity, and approximately 80 µL of endometrial fluid was gradually removed with a syringe. Once the sample was collected, suction was stopped, and the catheter was removed; (3) endometrium, a flexible sterile cannula or catheter was inserted through the cervix under ultrasound guidance into the uterus. Once in contact with the uterine wall, a suction was performed to biopsy the endometrial tissue. It is important not to perform any suction until you are sure that you have reached the endometrial wall and not to suction immediately after obtaining the tissue to avoid contamination of the sample with other fluids. In some cases, it may be necessary to clamp the cervix with forceps; (4) intestinal, a sample was taken for the rectal area with the help of a sterile swab by inserting the swab at 10 cm and rotating for 30 s; and (5) oral, with a sterile swab rotating for 60 s around the mucosa. Then, they were stored at −80 ° C, correctly identified, and grouped in boxes according to sample type until DNA extraction and sequencing were performed.

### 2.3. DNA Extraction and Sequencing

Microbial DNA was extracted from the samples using the QIAamps Fast DNATissue Kit (Qiagen, Hilden, Germany) following the manufacturer’s instructions. 16S rDNA regions were PCR amplified using the Ion 16S™ Metagenomics Kit (Thermo Fisher Scientific, Waltham, MA, USA). The resulting amplicons were quantified using the Ion Universal Library Quantitation Kit (Cat. No. A26217) via qPCR. Subsequently, template preparation was conducted using the Ion PGM™ Hi-Q™ Template OT2 Kit (Thermo Fisher Scientific, Waltham, MA, USA) on the Ion OneTouch™ 2 System. Enriched Ion Sphere™ Particles were sequenced with the Ion PGM™ system (Thermo Fisher Scientific, Waltham, MA, USA).

### 2.4. 16S rRNA Data Analysis

We performed microbiome analysis using the QIIME2 bioinformatics platform (v2022.8) to process raw 16S rRNA gene sequences [33]. Sequences were quality filtered, denoised, and dereplicated using the DADA2 algorithm implemented in the denoise-pyro plugin, specifically designed for single-end demultiplexed pyrosequencing sequences [34]. To eliminate low-quality data, we trimmed the first 15 bases at the 5′ end of the sequences, and samples with a mean Phred quality score below 20 were excluded from further analyses. The obtained amplicon sequence variants (ASVs) were taxonomically classified using the Greengenes 13_8 99% Operational Taxonomic Units (OTUs) reference sequence database [35,36] with the VSEARCH tool [37]. To assess both alpha and beta diversities, a phylogenetic tree was constructed using Fasttree based on the alignment of ASV created with Mafft [38,39]. Diversity metrics, including Faith’s phylogenetic diversity, UniFrac distance, Jaccard distance, and Bray–Curtis dissimilarity, were estimated using the diversity core-metrics-phylogenetic plugin at a sampling depth of 3400 sequences per sample. 

### 2.5. Statistical Analysis

We examined and statistically analyzed the microbiome data obtained using R v.4.2.2 [40]. To evaluate differences in alpha diversity between sample types and across body tracts, we used the pairwise Wilcoxon rank sum test adjusted by the Benjamini–Hochberg procedure. For comparing beta diversity among the different body tracts, we utilized pairwise Permutational Multivariate Analysis of Variance [41] with Bonferroni correction for multiple comparisons. These methods offer robust statistical approaches to evaluate diversity patterns and differences in microbiome composition within and between sample categories. We defined significance at *p* < 0.05.

To gain a deeper understanding of the factors that impact microbial diversity and the dynamics of the female microbiome ecosystem concerning infertility, we performed a nestedness test [42] in all types of collected samples. This statistical approach allowed us to identify potential patterns of genera distribution, in which the microbiota present in more diverse samples was also observed in less diverse samples. The nestedness analysis aimed to provide information on the interrelationships and potential ecological connections between microbial communities within the context of infertility studied.

## 3. Results

### 3.1. Cohort Description

**Figure 1:**
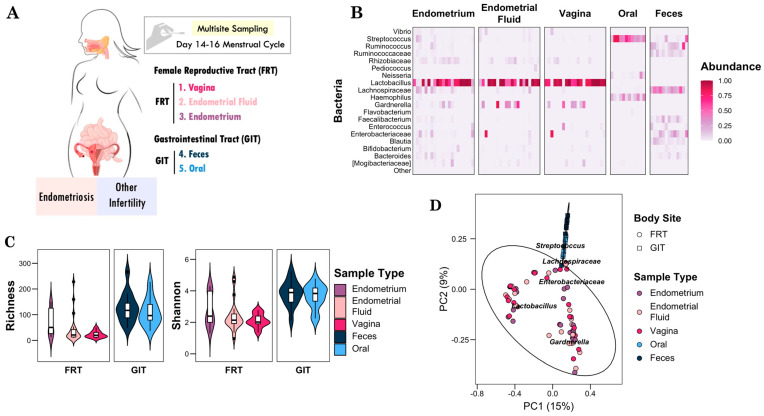
Female gastrointestinal and reproductive microbial profile. (**A**) Diagram that includes the body sites of the female reproductive tract (FRT) and the gastrointestinal tract (GIT). The types of samples collected for the study in the GIT were oral and feces and in the FRT were endometrium, endometrial fluid, and vagina. (**B**) Relative abundance of the main taxa in the sample types included in the study. Each column corresponds to an individual sample, and the color shows relative abundance. (**C**) Violin plot showing richness and Shannon indices as alpha diversity. The panels were separated by body sites. The sample types are shown as variables within each body site. (**D**) Principal coordinate analysis using Bray–Curtis distances for the distribution of the microbial community between body sites. Each point corresponds to a sample. The shapes show the body sites and colors for the sample types included in the study. Ellipses represent aggregation by body sites.

The pilot study was a cross-sectional cohort of women affected by infertility from different causes. A total of 21 patients were available for analysis. Sampling of the GIT as a distant microbial axis included oral and intestinal samples. The FRT as a local microbiome of infertility is represented by the vagina, endometrial fluid, and endometrium (Figure 1A). Therefore, we analyzed the microbiome of a total of 105 samples. Furthermore, we collected data associated with demographic and medical status as shown in Table 1. We grouped the participants into gynecological conditions associated with infertility in endometriosis and other conditions. The endometriosis group consisted of 8 women (38.09%), while the other conditions group included 13 women (61.90%) diagnosed with different infertilities, such as ovarian failure, polyp, erythroplakia, hydrosalpinx, chronic endometritis, or polycystic ovary syndrome.

### 3.2. Female Holobiont as a Gastrointestinal and Reproductive Microbial Profile

To assess the female microbial population, we analyzed body sites from both the FRT and GIT. We characterized the bacterial composition of samples taken from the endometrium, endometrial fluid, vagina, oral cavity, and feces. In the FRT samples, there was an average *Lactobacillus* abundance of 67.03%. This predominant genus was consistent across the FRT sites, with figures of 63.2% in the endometrium, 63.64% in the endometrial fluid, and 75.29% in the vagina. However, some FRT samples showed opposite trends between *Lactobacillus* and *Gardnerella*, suggesting that a particular microbial interaction in a certain woman could be related to infertility. In contrast, distant microbial niches such as oral and fecal exhibited a typical microbial composition of the GIT. The bacterial population of the oral microbiome differed markedly from that of the FRT microbiome, with a higher abundance of *Streptococcus*, *Haemophilus*, and *Neisseria*. The fecal microbiota showed a more diverse population, highlighting the presence of *Streptococcus*, *Ruminococcus*, *Lachnospiraceae*, and *Enterobacteriaceae* (Figure 1B). Interestingly, we identified *Lachnospiraceae* and *Enterobacteriaceae*, typically associated with the intestinal niche, in the endometrium when *Lactobacillus* levels were low (<25%), suggesting possible translocation and antagonisms between microorganisms from distant body sites.

We determined microbial diversity by examining the bacterial richness and diversity at each site in the body. For this purpose, we explored the alpha diversity of each sample type using the richness and Shannon indices. Richness was defined by the number of amplicon sequence variants (ASVs) observed, which can be interpreted as the count of distinct bacterial taxa. For the FRT, the richness was 44.7, while for the GIT, it was 119.8. Consequently, the FRT samples had significantly lower richness than the GIT samples (Figure 1C left panel, *p* = 6.8 × 10^−8^). The value of microbial diversity for the FRT community was 2.4, suggesting that it was dominated by a few bacterial genera. On the contrary, the diversity value for the GIT site was 3.7, which shows that the gastrointestinal site was populated with a more diverse community. Therefore, we found statistical differences between the FRT and GIT body sites (Figure 1C right panel, *p* = 1.3 × 10^−7^). Although the tracts exhibited differential alpha diversity, the sample types within each tract showed a similar distribution, supporting niches of the same body site that could be adapted to specific requirements such as the gastrointestinal and reproductive environments. 

Next, we estimate the dissimilarity between sample types through a beta diversity analysis (Figure 1D). Using the Bray–Curtis index, we quantified the compositional dissimilarity between sites by comparing genera abundances. We color-coded the five sample types from the FRT and GIT to visualize their distribution in a Principal Correspondence Analysis. The results showed that the GIT samples clustered distinctly from the FRT samples (*p* = 1 × 10^−3^, PERMANOVA). Within the FRT sample distribution, we identified two groups based on whether the community was dominated by *Lactobacillus* or *Gardnerella* abundance. The lack of separation among the types of FRT samples suggests that clinical characteristics could influence the subtypes of the microbial community within the FRT. We noted that the FRT and GIT exhibited distinct microbial compositions and distributions, indicative of niche specificity.

### 3.3. FRT–GIT Microbiome Connection in Infertility through Nestedness

**Figure 2:**
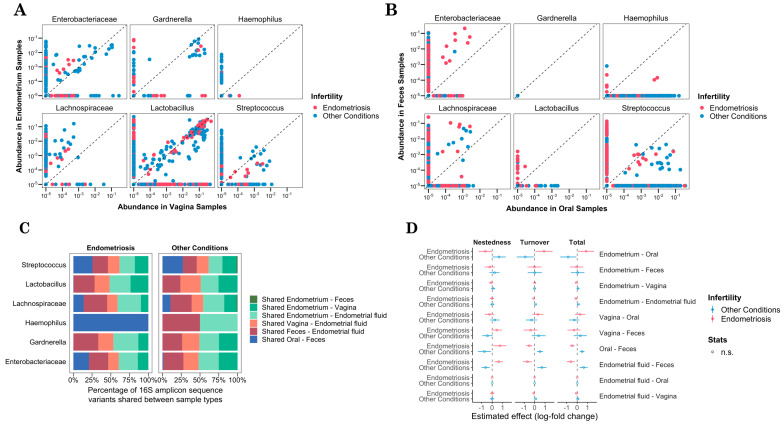
FRT–GIT microbiome connection in infertility through nestedness. (**A**) Top six common bacterial ASVs between endometrial and vagina samples as female reproductive tract sample types, (**B**) and ASVs between oral and fecal samples corresponding to the gastrointestinal tract. (**C**) Relative abundance of different ASVs found in each group when compared between different body sites in percentages. ASV, amplicon sequence variant. (**D**) Nestedness (richness difference between sites), turnover (genera replacement between sites), and total microbial taxa comparing endometriosis and other conditions.

To determine whether bacterial taxa were shared or sample type-specific, we analyzed nestedness between niches. Specifically, we evaluated the distribution of amplicon sequence variants (ASVs) among sample types within infertility groups. For that, we analyzed the variability of all ASVs or inferred DNA sequences attributed to bacterial taxa for each sample type. Therefore, we obtained the shared ASVs for pairwise sample types, represented along the diagonal dashed line as shown in Figure 2A, and the unique ASV for a specific sample type, located on a particular side under the dashed line. The study groups were color-coded for differentiation. Focusing on the top six bacterial taxa, we found that, in both study groups, *Lactobacillus* was the most shared ASV in the vagina and endometrium as the FRT sample types (Figure 2A, middle panel). *Gardnerella* and *Enterobacteriaceae* were mainly detected in other gynecological conditions, but not in cases of endometriosis. In particular, the only *Gardnerella* ASV shared between endometriosis the FRT sites belonged to a specific participant (Figure S1). These results associated with infertility groups might explain the differences observed in the subtypes of the microbial community, as previously found in Figure 1D. In the shared microbiome of the GIT sites, ASVs corresponding to *Streptococcus* were mainly identified in oral samples related to other conditions (19 ASVs). On the contrary, *Enterobacteriaceae* present in fecal samples were associated with participants with endometriosis (7 ASVs) (Figure 2B).

After analyzing the female microbiome, we showed that an increase in *Enterobacteriaceae* and *Lachnospiraceae* appeared to be associated with a decrease in *Lactobacillus* in the endometrial microbiome (Figure 1B). Therefore, the bacterial ASVs found within the FRT and GIT sites related to the infertility groups raise the question of whether the body sites shared the microbiome. We analyzed the shared ASVs between body sites and sample types (Figure 2C). We observed higher levels of shared *Enterobacteriaceae* (25%) and *Gardnerella* (24.61%) in endometrial and vaginal samples for women with other conditions compared to those with endometriosis (13.95% and 13.33%, respectively). In contrast, higher levels of shared *Enterobacteriaceae* (20.93%) were found in oral–fecal samples of women with endometriosis compared to other conditions of infertility (0.96%). *Gardnerella* appeared in both the FRT and FRT–GIT comparisons but was absent in the GIT-only samples in both study groups. In oral and fecal samples, the relative abundance of *Streptococcus* ASV was elevated in both study groups, 25.64% for women with endometriosis and 27.15% for those with other conditions. It also should be noted that *Haemophilus* was identified as a specific taxon associated with the GIT in cases of endometriosis. The diversity of ASVs within *Haemophilus* was limited to two distinct ASVs (Figure 2B). In other infertility conditions, *Haemophilus* was present in endometrium–endometrial fluid and feces–endometrial fluid comparisons. This finding could explain the ability of *Haemophilus* to adapt to the endometriosis environment, compared to other conditions of infertility.

The ASVs of *Lachnospiraceae* and *Streptococcus* found in the endometrium were similar but not identical to those found in the fecal samples, suggesting a past microbial transfer or possible contamination between the anus and vagina (Figure S2C). Importantly, we did not find additional shared bacterial ASVs in endometrial–fecal comparisons, which supports the notion of niche specificity within the FRT and GIT and rules out the possibility of cross-contamination during the sampling. No significant microbial overlap was detected between the endometrial and oral samples, nor between the vaginal and oral or fecal samples (Figure S2).

We profiled the microbial community using beta diversity metrics such as nestedness, which measures richness differences between sites and turnover, which captures genera replacement between sites [29]. We conducted pairwise comparisons of sample types, considering that an estimated nestedness value of 0 would indicate a high degree of microbiome sharing, as evidenced by similar ASV profiles. The turnover, which reflects changes in genera composition or ASVs due to environmental dissimilarities and spatial distance between body sites, served as a complementary measure to the nestedness. An estimated turnover effect closer to 0 indicated a minimal change in genera composition between sites (Figure 2D). We did not observe a significant estimated effect among the FRT-related sample types, i.e., endometrium, endometrial fluid, and vagina, suggesting homogeneity in ASV. On the contrary, we found dissimilarity in GIT sample types, but not a significant one, suggesting that oral and fecal niches may have different environments, possibly due to their distant body locations. In addition, the comparison of the paired samples from the FRT and GIT revealed dissimilarities in their microbial communities. We did not find a significant number of shared ASVs between the FRT and GIT sites concerning the infertility groups. Thus, the FRT–GIT body sites did not seem to share the main bacterial communities. Therefore, balance within the FRT or GIT sites may be essential to maintain infertility-associated homeostasis. Although the FRT and GIT do not exchange microorganisms, the imbalance of one of them could potentially affect the overall microbial ecology and impact female fertility.

### 3.4. Female Reproductive Tract Microbiome Associated with Infertility

**Figure 3:**
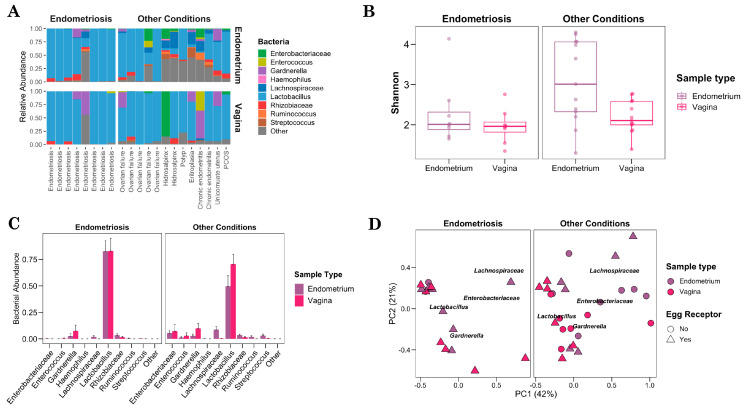
Female reproductive tract microbiome associated with infertility. (**A**) Main relative abundance of bacteria showing the variability between patients (in columns) separated by endometriosis and other infertility conditions (vertical panels) and sample types such as endometrium and vagina (horizontal panels). (**B**) Boxplot showing the diversity index for sample types (endometrium and vagina) and infertility groups such as endometriosis and other infertility conditions. (**C**) Mean of bacterial abundance and its standard deviation related to endometriosis and other infertility conditions. (**D**) Principal coordinate analysis that evaluates the distribution of the microbial community using weighted Unifrac distances. The colors correspond to the type and shape of the sample to women as an egg receptor.

Focusing on the FRT sample types, we evaluated the bacterial composition and diversity of endometrial and vaginal samples for the gynecological condition that underlies infertility. Our findings revealed a high degree of homogeneity between endometriosis samples and a striking similarity between the endometrial and vaginal microbiomes. The low diversity of genera observed in these samples, along with the predominance of *Lactobacillus*, suggests a distinctive microbial profile associated with endometriosis. On the contrary, endometrial samples from women with infertility conditions other than ovarian failure exhibit significant differences in microbial composition and abundance (Figure 3A). Therefore, intraindividual and interindividual variability among samples hinders the establishment of a specific microbial pattern associated with infertility. However, it is noteworthy that, within this group, patients diagnosed with ovarian failure had a *Lactobacillus*-dominated microbiome, similar to the FRT microbiome of endometriosis. These findings suggest that the composition of the microbiome in women with infertility can vary widely and may be more closely related to the specific gynecological condition.

To explore the endometrial and vaginal diversity in the infertility condition, we used the Shannon index. Interestingly, women with endometriosis showed significantly lower bacterial diversity compared to those with other infertility conditions (*p* = 0.027). The mean diversity of endometrial samples (Shannon index: 2.29) closely resembled that of vaginal samples (Shannon index: 1.97) in women with endometriosis. In contrast, women with other infertility conditions exhibited a significantly more diverse bacterial population in the endometrium compared to the vagina (*p* = 0.027, Wilcoxon) (Figure 3B). Then, we assessed the bacterial abundance of the main taxa identified in endometrial and vaginal samples across the studied groups. In women with endometriosis, both types of samples were predominantly dominated by *Lactobacillus*, with an average abundance of 82.63% in endometrial samples and 82.93% in vaginal samples. Although women with other infertility conditions showed a more diverse microbiome, *Lactobacillus* remained the most abundant taxon in both types of samples. Although *Lactobacillus* represented less than half of the bacterial population in the endometrial microbiome (49.62%), it represented a higher dominant proportion of 70.54% in vaginal samples (Figure 3C). However, there were no significant differences in *Lactobacillus* abundance between the endometrial and vaginal samples. These findings suggest that the inflammatory environment inherent in endometriosis may promote the survival of specific acidophilic genera, such as *Lactobacillus*, leading to an imbalance in the microbiota of the female reproductive tract.

To evaluate the influence of infertility conditions on the structure and composition of the microbial community, we used weighted UniFrac distances. The principal coordinate analysis was performed considering the clinical variable of egg reception. Consistent with our previous statistical analyzes, endometrial and vaginal samples from women with endometriosis exhibited similar distributions (*p* = 0.624, PERMANOVA), without microbiota-related clusters associated with the type of FRT sample. On the other hand, endometrial samples from women with other infertility conditions exhibited a distinct separation from vaginal samples (*p* = 0.007, PERMANOVA). Interestingly, the majority of women with endometriosis (7 out of 8) and other women with ovarian failure, endometrial polyp, or fallopian tubes problem (such as hydrosalpinx) that had an FRT profile similar to endometriosis required egg donation for a successful pregnancy (Figure 3D and Figure S4). In contrast, women who did not require egg donation had a distinctive endometrium and vaginal microbiome. Our results suggest that pathologies that compromise ovarian function and ovulation could affect the fertility-related microbiota in the reproductive tract, potentially leading to difficulties in achieving pregnancy.

## 4. Discussion

The microbiota lives in symbiosis with the human body. The microbial population is adapted to environmental conditions, such as hormones, allowing certain genera to colonize a particular body site. Endometriosis is an estrogen-dependent infertility-related disease that affects the female reproductive tract and its resident microbiota. Here, we investigated the FRT–GIT microbiome associated with endometriosis and other infertility conditions. We showed that the endometrium and vagina niches did not have a differential microbial community in endometriosis. Specifically, we found a *Lactobacillus*-dominant microbiome in endometriosis like the diagnosis of ovarian failure, but more diverse in other infertility conditions. This similarity in the female holobiont context may be attributable to the hormonal environment. Endometriosis, in its initial stages, could enhance fertility due to abnormally high estrogen levels. However, over time, this overproduction can negatively affect the female reproductive system, impeding its normal function [4]. In contrast, ovarian failure, characterized by insufficient estrogen production, is clinically managed with hormone replacement therapy. Thus, it can be stated that estrogen dysregulation affects the FRT microbiome and consequently impacts fertility. On the other hand, diverse microbial communities in the FRT and other sites of the distal body, such as the GIT, were associated with infertility-associated conditions. Ultimately, the GIT microbiome probably also undergoes changes due to environmental modifications associated with hormones, as observed in endometriosis, showing a distal microbial modification. 

The holobiont is a biological system made up of a host organism and all its associated microbes. Within an ecological niche, these multiple microorganisms are interconnected and interdependent [2,43]. In our study, we evaluated the body sites of the FRT–GIT with histological and physiological differences that affect its resident microbiome. We found specific microbial communities belonging to each tract. Other studies investigated microbial communities at multiple FRT body sites related to infertility, such as the peritoneum and feces, but inconsistent results have been derived due to variations in clinical characteristics or methods [13,44]. However, no studies have included the oral microbiome to evaluate endometriosis and other conditions associated with infertility. However, increasing evidence suggests that periodontal disease and female infertility conditions could be associated [45,46,47]. Although the host–microbial balance may be directly affected in the uterus of women with endometriosis. Therefore, the holobiont perspective could investigate the impact on other body sites and how it is related to the disease.

We found that the *Lactobacillus* genera dominated the FRT microbiomes under conditions of endometriosis and ovarian failure. On the contrary, numerous studies linked *Lactobacillus* to a healthy status [48,49,50]. The microbial profile could change depending on the endometriosis stage, where the early stages preserve the *Lactobacillus* populations, while the later stages were more diverse [51]. Wessels and Cols. found a higher proportion of other bacteria that differed from *Lactobacillus* in patients with stage 4 endometriosis compared to the control in the endometrial biopsy tissue [52]. Furthermore, the intestinal microbiome as a distal body site also varies in endometriosis stages [53]. Assessing the complexity of chronic diseases such as endometriosis is a challenge for physicians, especially when undergoing fertility treatment. Therefore, variation in the stage, niche, or heterogeneous clinical methods of endometriosis for bacterial identification could explain the inconsistency of the role of *Lactobacillus* in endometriosis.

Recent studies have pointed to endometrial infection by *Fusobacterium* as a mechanism for the pathogenesis of endometriosis. However, only 64.3% of the endometrial samples from women with endometriosis that they examined tested positive for *Fusobacterium* [12]. In our cohort, 25% of women with endometriosis and 53.84% of women with another infertility condition had *Fusobacterium* present in their endometrial samples (Figure S3). The observed discrepancy in the prevalence of *Fusobacterium* could be attributed to our limited sample size and the inherent biological variability among the studied populations. Furthermore, the fact that both studies observed women with endometriosis without this genus in their endometrial samples suggests that other individual factors, such as diet, lifestyle, medication use, and medical history, have the potential to significantly influence the composition of the endometrial microbiome.

We determined the endometriosis microbial taxa shared or unique in the FRT–GIT compared to other conditions associated with infertility. Nestedness can have implications for ecosystem stability, resilience, and function [54,55,56]. *Lactobacillus* ASV, the most abundant bacteria found in vaginal and endometrium samples, was shared independently of the infertility condition. In contrast, *Gardnerella* was found in the FRT samples for all patients but shared ASV distributed differently for other conditions associated with infertility while being site-specific for patients with endometriosis [51,57]. One of the most relevant results of this study is the presence of *Haemophilus* in oral samples from patients with endometriosis. *Haemophilus* is a dominant bacterium in the microbiota of asthmatic children [58,59,60,61,62,63,64]. Recently, a comparative study of the oral microbiota of patients with asthma treated and not treated with steroids revealed that *Haemophilus* was one of the dominant genera in patients not treated with steroids [65], which may be related to the fact that endometriosis is an estrogen-dependent disease (a type of steroid produced by the ovary). An effective treatment to maintain a vaginal microbiota dominated by *Lactobacillus*, beneficial for in vitro fertilization (IVF) treatments, involves maintaining stable estrogen levels. An association between *Haemophilus* and endometriosis was observed, along with its potential relationship with estrogen levels and hormonal treatment [66,67]. On the other hand, *Haemophilus influenzae* has been associated with the etiology of periodontitis [68], a condition frequently found in patients with endometriosis. The finding suggests that endometriosis might have environmental conditions corresponding to outer and inner FRT sites that limit colonization for some microbial genera. This could impact the dynamics of the ecosystem, especially in response to disturbances or changes in environmental conditions.

The findings of this study should be considered despite some limitations. The study had a reduced sample size or lacked healthy controls that included only eight patients with endometriosis and the rest with other conditions associated with infertility. However, the evaluation of the microbiome in multiple sample types per patient showed the complexity of the intraindividual microbiome [69]. Furthermore, we understand that the sex hormone might be a modulator of the host–microbe interaction at a particular body site [70]. The samples in our cohort were obtained from women before undergoing fertility treatment. Because the date for microbiome sampling did not match the hormonal panel analysis, we opted not to include data that would not represent the hormonal environment of the microbiome. Future research should consider the potential effects of local hormonal levels in fluids related to the site-specific microbiome. 

## 5. Conclusions

In summary, our study advances our understanding of the role of the female microbiome in infertility, offering new research possibilities and potential strategies for infertility prevention and treatment. We found that while endometrial and vaginal microbiomes exhibit similarities in endometriosis cases, a dominance of *Lactobacillus* is observed, resembling microbiomes seen in ovarian failure. However, other infertility conditions show more diverse microbial communities. Additionally, associations between microbial communities in the female reproductive tract and other body sites suggest broader hormonal influences. Notably, we identified a potential link between the presence of *Haemophilus* in oral samples from patients with endometriosis and hormonal environments. Despite limitations such as a small sample size, our findings underscore the importance of considering hormonal influences on microbial dynamics in infertility-related conditions. Collectively, we propose a female holobiont applied to microbial ecology that focuses on the importance of the balance of female sex hormones in the host–microbial interaction in health and disease. This insight could lead to innovative approaches to infertility management. However, more research is essential to establish causal links between microbiome variations and infertility and to identify potential therapies to restore microbial balance and enhance fertility.

## Figures and Tables

**Table 1:** Clinical and demographic characteristics of the participants. Type of blood from 2 unregistered patients (other conditions group). Contraceptive use of 1 unregistered patient (endometriosis group). Alcohol and tobacco use of 1 unregistered patient (other conditions group).

Gynecological Condition	Age (Mean)	Autoimmune Disease	Egg Receptor	Contraceptives	Eutirox	Other Drugs	Alcohol Habit	Tobacco Habit
Endometriosis (8)	42.7 ± 5.5	2 (25%)	7 (87.5%)	4 (57.1%)	2 (25%)	2 (25%)	2 (25%)	1 (12.5%)
Other conditions (13)	39.4 ± 3.7	1 (7.69%)	6 (46.15%)	11 (84.6%)	3 (23%)	4 (30.7%)	4 (33.3%)	0 (0%)

## Data Availability

16S rRNA sequencing data have been deposited at the NCBI database and are publicly available as of the date of publication with accession number PRJNA1031834. All original codes have been deposited at Zenodo and are publicly available at 10.5281/zenodo.10362432. Any additional information required to reanalyze the data reported in this paper is available from the corresponding author upon request.

## Supplementary Materials

The following supporting information can be downloaded at: https://www.mdpi.com/article/10.3390/microorganisms12050989/s1, Figure S1. ASV shared between endometrial and vaginal bacteria by subject. The six most common bacterial ASVs between endometrial and vagina samples presented for (A) women with endometriosis and (B) those with other infertility conditions. ASVs are color-coded by subject, facilitating the identification of ASVs shared by the same participant. Figure S2. ASV shared between sample types by infertility-associated condition. Six main common bacterial ASVs between (A) endometrial and endometrial fluid samples, (B) endometrial and oral samples, (C) endometrial and feces samples, (D) endometrial fluid and vagina samples, (E) endometrial fluid and oral samples, and (F) endometrial fluid and feces samples, (G) vagina and oral samples, (H) vagina and feces samples. ASVs are color-coded to represent infertility conditions: red for endometriosis and blue for other conditions. Figure S3. Distribution of Fusobacterium proportions in endometrial, oral, and feces samples according to infertility-associated condition. Figure S4. Microbial community distribution of vaginal and endometrial samples. Principal coordinate analysis that evaluates microbial community distribution using weighted Unifrac distances. The colors represent the clinical diagnosis, and the shape corresponds to women as egg receptors.
