# Supplementary material for: Distinct Gastrointestinal and Reproductive Microbial Patterns in Female Holobiont of Infertility
Source: Microorganisms. 2024 May 14;12(5):989. doi: 10.3390/microorganisms12050989 (PMC11124254; doi:10.3390/microorganisms12050989)
Supplement: Supplementary file 1 [file microorganisms-12-00989-s001.zip › microorganisms-2984280-supplementary.pdf]

SUPPLEMENTARY FIGURES

Distinct Gastrointestinal and Reproductive Microbial Patterns in Female Holobiont of Infertility

Ana T Marcos[1,2†], María J Rus[3†], Victoria Areal-Quecuty[3], Aurea Simon-Soro[3*], José Manuel Navarro-Pando[1,2]

[1]Cátedra de Reproducción y Genética Humana del Instituto para el Estudio de la Biología de la Reproducción Humana (INEBIR), Universidad Europea del Atlántico (UNEATLANTICO) - Fundación Universitaria Iberoamericana (FUNIBER), Sevilla, 41001, Spain.

[2]Hospital San Juan de Dios Sevilla, 41005, Spain.

[3]Departamento de Estomatología, Facultad de Odontología, Universidad de Sevilla, Sevilla, 41009, Spain.

†These authors contributed equally.

*Correspondence author: asimon@us.es (AS-S)

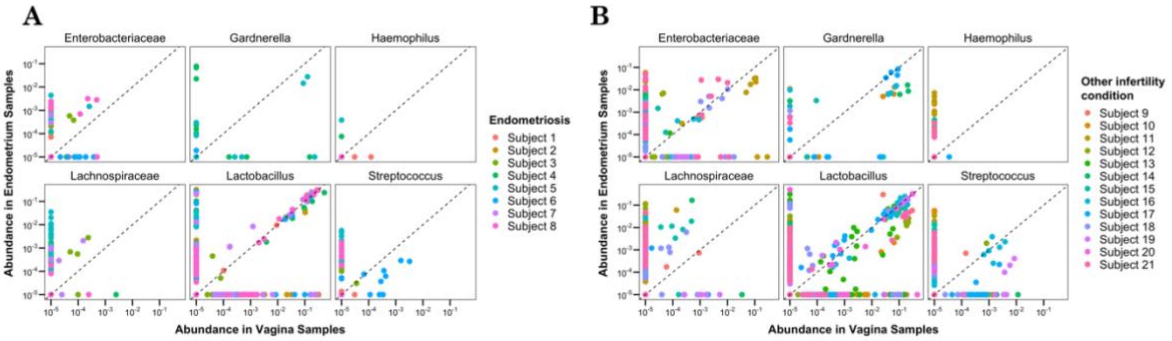

Figure S1. ASV shared between endometrial and vaginal bacteria by subject. The six most common bacterial ASVs between endometrial and vagina samples presented for (A) women with endometriosis and (B) those with other infertility conditions. ASVs are color-coded by subject, facilitating the identification of ASVs shared by the same participant.

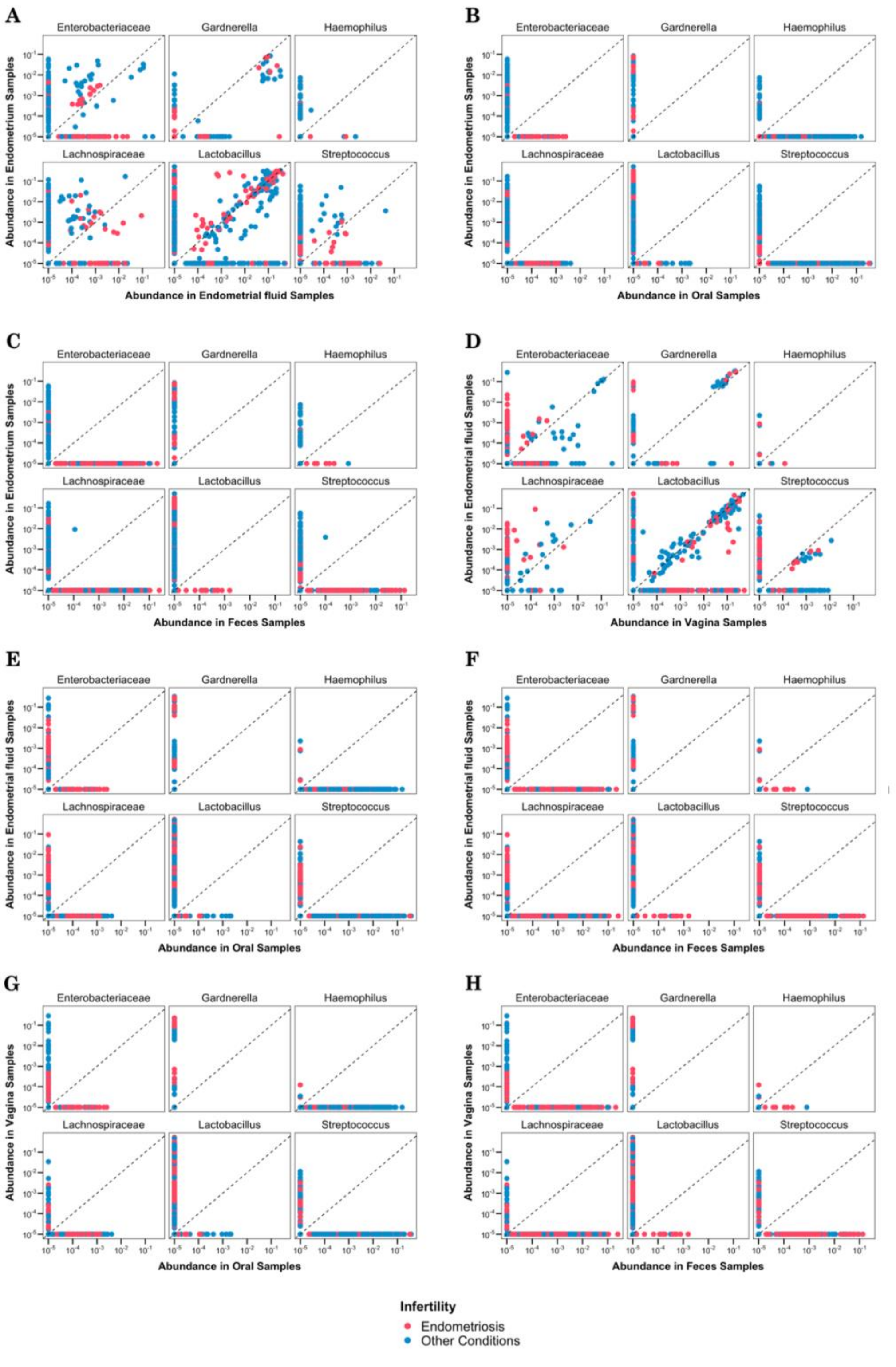

Figure S2. ASV shared between sample types by infertility-associated condition. Six main common bacterial ASVs between (A) endometrial and endometrial fluid samples, (B) endometrial and oral samples, (C) endometrial and feces samples, (D) endometrial fluid and vagina samples, (E) endometrial fluid and oral samples, and (F) endometrial fluid and feces samples, (G) vagina and oral samples, (H) vagina and feces samples. ASVs are color-coded to represent infertility conditions: red for endometriosis and blue for other conditions.

Figure S3.

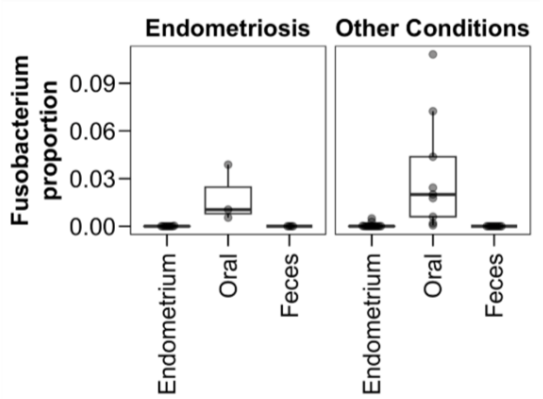

Distribution of *Fusobacterium* proportions in endometrial, oral, and feces samples according to infertility-associated condition.

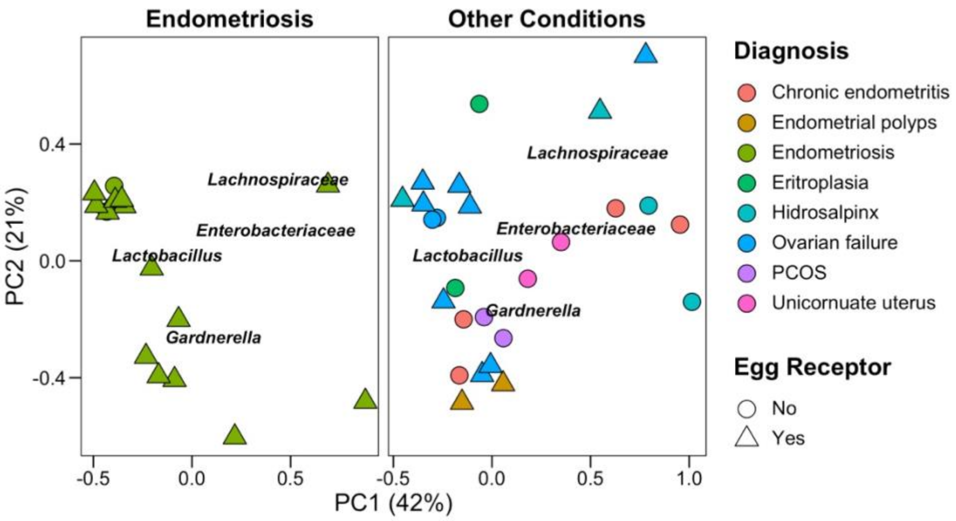

Figure S4. Microbial community distribution of the vaginal and endometrial samples. Principal coordinate analysis that evaluates the distribution of the microbial community using weighted Unifrac distances. The colors represent the clinical diagnosis, and the shape corresponds to women as egg receptors.
